# Quality assessment of videos on social media platforms related to gestational diabetes mellitus in China: A cross-section study

**DOI:** 10.1016/j.heliyon.2024.e29020

**Published:** 2024-03-31

**Authors:** Qin-Yu Cai, Jing Tang, Si-Zhe Meng, Yi Sun, Xia Lan, Tai-Hang Liu

**Affiliations:** a The Joint International Research Laboratory of Reproduction and Development, Chongqing Medical University, Chongqing, 400016, China; b Department of Obstetrics and Gynecology, Women and Children's Hospital of Chongqing Medical University, Chongqing, 400037, China; c Department of Bioinformatics, The School of Basic Medicine, Chongqing Medical University, No.1 Yixueyuan Rd, Yuzhong District, Chongqing, 400016, China

**Keywords:** Gestational diabetes mellitus, Social media platform, Quality, Medical education, Health information

## Abstract

**Purpose:**

This study aimed to systematically evaluate the quality of content and information in videos related to gestational diabetes mellitus on Chinese social media platforms.

**Methods:**

The videos on various platforms, TikTok, Bilibili, and Weibo, were searched with the keyword “gestational diabetes mellitus" in Chinese, and the first 50 videos with a comprehensive ranking on each platform were included for subsequent analysis. Characteristic information of video was collected, such as their duration, number of days online, number of likes, comments, and number of shares. DISCREN, JAMA (The Journal of the American Medical Association) Benchmark Criteria, and GQS (Global Quality Scores) were used to assess the quality of all videos. Finally, the correlation analysis was performed among video features, video sources, DISCERN scores, and JAMA scores.

**Results:**

Ultimately, 135 videos were included in this study. The mean DISCERN total score was 31.84 ± 7.85, the mean JAMA score was 2.33 ± 0.72, and the mean GQS was 2.00 ± 0.40. Most of the videos (52.6%) were uploaded by independent medical professionals, and videos uploaded by professionals had the shortest duration and time online (P < 0.001). The source of the video was associated with numbers of “likes", “comments", and “shares" for JAMA scores (P < 0.001), but there was no correlation with DISCERN scores. Generally, videos on TikTok with the shortest duration received the most numbers of “likes", “comments", and “shares", but the overall quality of videos on Weibo was higher.

**Conclusion:**

Although the majority of the videos were uploaded by independent medical professionals, the overall quality appeared to be poor. Therefore, more efforts and actions should be taken to improve the quality of videos related to gestational diabetes mellitus.

## 1 Introduction

Gestational diabetes mellitus (GDM) significant complication during pregnancy, affecting approximately 15% of pregnant women globally, and it increases the risk of various short-term and long-term issues for both maternal and fetal health, including obesity, impaired glucose metabolism, and cardiovascular disease [[1], [2], [3], [4]]. Prenatal management of pregnant women with GDM, involving a combination of dietary control, regular exercise, blood glucose monitoring, and medication, is typically concentrated within a relatively short timeframe during pregnancy and is primarily overseen by healthcare professionals in the healthcare system. However, the time and place restrictions often hinder patient compliance with GDM management, thus placing an additional burden on the healthcare system [5,6]. Thus, it is essential to recognize that the management of diabetes during pregnancy should not solely rely on the healthcare system [7,8]. Improving prenatal self-education and awareness among pregnant women with GDM is crucial. The ever-increasing development of internet technologies offers patients more opportunities to access health information and engage in health communication [9,10]. It is common for pregnant women, particularly younger women, to turn to the internet, social media platforms, and smartphone apps for information during their pregnancies [11,12]. This trend is particularly prevalent among women aged 19 to 35 in China [13]. Web searches cover a wide range of pregnancy-related topics, including fetal development, maternal complications, prenatal care, medication safety, nutrition, and mental health during pregnancy [14]. Searches for health information related to the prenatal, perinatal, and postnatal periods are on the rise, and more information is becoming available online [15,16]. Despite the wealth of digital resources on social media platforms, the absence of regulations or guidance regarding video content can result in misleading and incomplete information [17]. Such inaccuracies and gaps in information can add to the burden of self-education and self-management for the general public's health. Hence, there is a need for quality standards and guidance on social media platforms. To the best of our knowledge, there is limited prior literature that focuses on GDM-related video content in China or the United States. For instance, Kong W et al. reviewed 199 TikTok videos using the coding schema proposed by Goobie et al. and DISCERN criteria. They found that the overall quality of diabetes-related information on TikTok is acceptable, but it might not fully meet the informational needs of patients with diabetes [18]. Similarly, Birch EM evaluated 115 unique YouTube videos on GDM in April 2020 using DISCERN and GQS. While some high-quality videos were found, the overall reliability, accuracy, and comprehensiveness were low, and higher quality did not correlate with increased viewer interaction [19]. These studies have produced inconsistent conclusions regarding video quality, and there is still a lack of knowledge regarding the content and quality of information about GDM on social media platforms in China.

In China, a multitude of social media platforms are available, but TikTok, Bilibili, and Weibo have emerged as the most popular and essential platforms for Chinese people to access and share health-related information [[20], [21], [22]]. These platforms enable individuals to conveniently access real-time information through computers, smartphones, and tablets [23,24]. While there is a wealth of health-related videos on these platforms, there remains a notable absence of quantitative assessments of the quality of content pertaining to health topics such as GDM, miscarriage, and preterm delivery. This study aimed to systematically assess the quality of content and information within GDM-related videos on these social platforms to provide evidence-based advice and recommendations to enhance the quality of information available.

## 2 Materials and methods

### 2.1 Search strategy and data collection

**Fig. 1:**
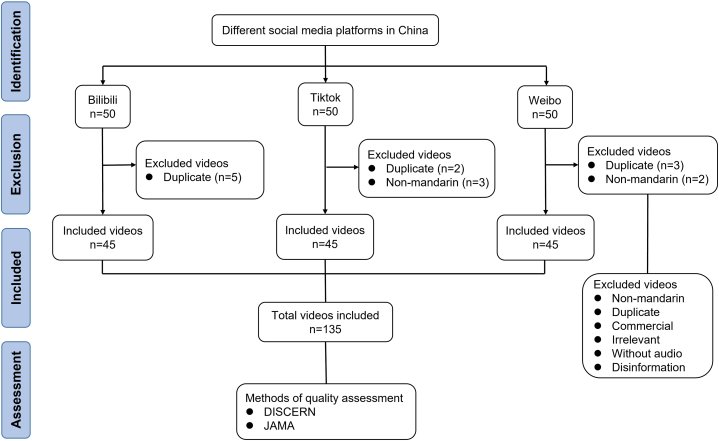
The flow chart of this study.

The Chinese keyword, gestational diabetes mellitus, was used to carry out a comprehensive search for popular science videos related to GDM on three Chinese social media platforms: Bilibili, TikTok, and Weibo. Our search encompassed all relevant content available up to October 31, 2022. To ensure impartiality in our search results and quality assessments, we employed newly created accounts on these platforms with no prior search history. The top 50 videos from each platform were selected based on their comprehensive ordering criteria, resulting in a total of 150 videos downloaded for our study. This sample size was determined to be adequate based on prior research [24,25]. Upon closer evaluation, we included videos directly related to GDM and excluded non-Mandarin videos, commercial content, videos lacking audio, duplicates, and videos containing disinformation. For consistency, we also excluded videos in languages other than Mandarin and any reposted videos. A detailed flowchart outlining the inclusion and exclusion criteria is manifested in Fig. 1.

The information regarding various video characteristics was gathered, including video duration, the number of days the video had been online, the number of likes, comments, shares, video types, and the region of the author. Additionally, the number of average daily likes, comments, and shares was automatically calculated. Based on prior research pertaining to the authors, we categorized video sources into six distinct types: independent medical professionals, patients or their guardians, medical institutions, news/media outlets, health educators, and independent non-medical users (non-medical professionals). Furthermore, video content was divided into four main categories: general information, education, daily sharing, and case discussion.

### 2.2 Assessment of quality

DISCREN, JAMA Benchmark Criteria, and GQS were used to conduct a quality assessment of the videos [[26], [27], [28]]. These tools have been widely employed in evaluating the quality of health-related videos on platforms such as YouTube, Facebook, and TikTok [29,30]. Additionally, for specialized medical aspects related to GDM, we employed the IADPSG/WHO 2010 criteria [31].

DISCERN is a comprehensive tool for assessing medical information and is among the most commonly used questionnaires. It comprises 16 aggregated questions, each scored on a scale ranging from 1 (poor) to 5 (good) points (Supplementary Table 1). All these 16 questions were divided into three sections. The first section, related to reliability, includes questions 1 to 8, addressing clarity of goals, relevance, and balance. The second section, questions 9 to 15, pertains to treatment, assessing how well it describes the workings of each treatment and illustrates its benefits, risks, and impact on quality of life. The final question, question 16, forms the third part, where users rate the overall quality of the publication as a source of information about treatment choices based on their responses to the previous questions. All videos were divided into five levels based on their total DISCERN scores, including very poor (<27), poor (27–38), fair (39–50), good (51–62), and excellent (63–75) [32]. The JAMA benchmark evaluates online information quality using four distinct criteria: authorship, attribution, disclosure, and currency [27]. Authorship necessitates that videos should include details about authors, contributors, and contact information, while attribution demands that references and sources should be listed properly. Disclosure requires conflicts of interest, financing, sponsorship, advertising, support, and video ownership should be disclosed and the requirement of currency is the dates the video was published and updated should be indicated. For each criterion, videos were rated as 0 if the criteria were not met and 1 if they were met. The total scores for these four criteria ranged from 0 to 4 (Supplementary Table 2). The GQS assesses educational value using 5 criteria (Supplementary Table 3). GQS scores range from 1 to 5, with a maximum score of 5 indicating high quality.

Two obstetricians, X L, and J T, independently evaluated all videos. Any differences or disputes between the two reviewers were resolved through discussion with the third author, Q C.

### 2.3 Statistical analyses

In subsequent statistical analysis, descriptive analysis of the data was conducted to provide an overview of both categorical and continuous variables. It's worth noting that none of the continuous variables followed a normal distribution. Nevertheless, we presented the continuous variables as both mean ± standard deviation (SD) and median with the interquartile range (IQR) of 25–75%, which offered a more detailed representation of the data. Additionally, categorical variables, such as the number of videos and video sources, were described in terms of frequencies and percentages. The equivalent nonparametric test, the Kruskal-Wallis H test, and the Bonferroni adjustment were used to analyze the differences between different groups, including video sources, DISCERN classification, and social media platforms. Correlations between the JAMA score, DISCERN score, and video features were calculated with Spearman's correlation coefficient. A P-value <0.05 was considered significant. Notably, no missing value in the variables used in the statistical analysis, and no extrapolation was made on the missing data in the analysis. All analyses were conducted using Statistical Package for the Social Sciences 26.0 (SPSS 26.0, IBM Corporation, Chicago, IL, USA) software.

### 2.4 Ethics Statement

This study concentrated on assessing the quality of videos contributed to and viewed by the public on social media platforms. It is important to note that no clinical data, human specimens, or laboratory animals were utilized in this research. All information used in this study was extracted from publicly available videos on Bilibili, TikTok, and Weibo, and no personal privacy data was involved in the process. Additionally, the study abstained from engaging in interactions with users, thereby eliminating the need for ethics committee approval.

## 3 Results

### 3.1 Video characteristics and quality assessment with DISCERN scores, JAMA, and GQS

**Fig. 2:**
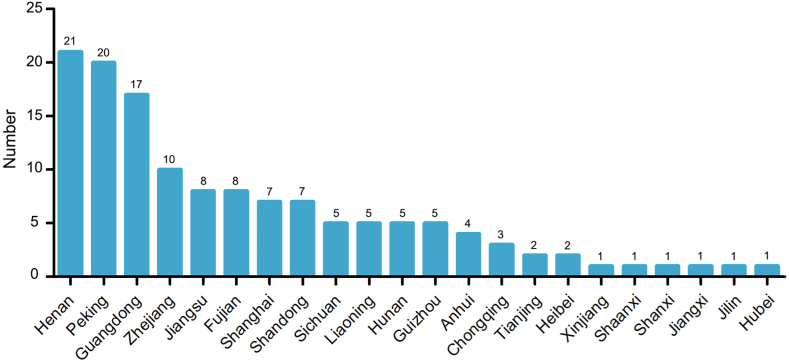
The distribution of video authors in China.

After screening the 150 videos, 10 duplicate and 5 non-mandarin videos were excluded, resulting in 135 videos being included eventually (Fig. 1). The mean DISCERN total score averaged 31.84 ± 7.85 (median (IQR): 30 (27, 34)), while the mean JAMA score was 2.33 ± 0.72 (median (IQR): 2 (2, 3); Table 1). However, GQS proved unable to provide a discriminating or accurate assessment of video quality. The majority of the videos (135 out of 145, or 93%) received a rating of 2, with the remainder being rated as 1 (3 out of 145, or 2%), 3 (4 out of 145, or 3%), or 4 (3 out of 145, or 2%). Consequently, GQS was excluded in the subsequent video quality and correlation analysis. The analysis of the regional distribution of authors indicated that the highest number of videos were uploaded from Henan, followed by Beijing and Guangdong (Fig. 2).

**Table 1:** Video characteristics of included videos.

Video characteristics	Mean ± SD	Median (IQR)
Duration (s)	241.26 ± 446.76	90 (58.5, 221)
Number of days online	412.23 ± 422.15	282 (85, 553.5)
Number of likes	2230.15 ± 7399.87	18 (1.5, 1189.5)
Number of likes/day	26.88 ± 149.53	0.07 (0.01, 5.12)
Number of comments	483.24 ± 1358.16	4 (0, 316.5)
Number of comments/day	4.71 ± 21.11	0.2 (0, 1.9)
Number of shares	521.04 ± 1441.42	14 (1, 355)
Number of shares/day	8.03 ± 57.73	0.5 (0.3, 1.7)
DISCERN reliability	18.03 ± 4.22	18 (15, 20)
DISCERN treatment	11.49 ± 4.21	10 (8, 13)
DISCERN quality	2.32 ± 0.79	2 (2, 3)
DISCERN total	31.84 ± 7.85	30 (27, 34)
JAMA score	2.33 ± 0.72	2 (2, 3)
GQS score	2.00 ± 0.40	2 (2, 2)

Further analysis of video sources revealed that most videos were uploaded by independent medical professionals (52.6%), followed by patients or their guardians (12.6%), medical institutions (11.9%), news/media (11.1%), health educators (8.1%), and independent non-medical users (3.7%). Interestingly, although videos uploaded by independent medical professionals had the shortest duration and days online (median (IQR): 65 (52, 110)), they appeared to be more popular, receiving the highest number of “likes" (median (IQR): 1180 (8,4392)), “shares" (median (IQR): 301 (2, 1082)), and “comments" (median (IQR): 262 (6930)) (Table 2).

**Table 2:** Video characteristics and quality assessment according to video sources.

Variable	Independent medical professionals	Patients or guardians	Medical institute	News/media	Health educators	Independent non-medica users	*P* value^b^
Numbers of videos^a^	71 (52.6%)	17 (12.6%)	16 (11.9%)	15 (11.1%)	11 (8.1%)	5 (3.7%)	–
Duration (s)	65 (52, 110)	278 (133, 533)	115 (65, 284)	70 (42, 110)	271 (160, 740)	199 (28, 233.5)	*P* < 0.001***
Number of days online	167 (56, 361)	834 (473, 1056)	268.5 (5.25, 515.5)	199 (45, 461)	492 (238, 697)	1361 (934.2, 1806.5)	*P* < 0.001***
Number of likes	1180 (8,4392)	36 (8, 95)	3 (0, 16)	4 (2, 61)	4 (2, 61)	2 (0, 14)	*P* < 0.001***
Number of likes/day	5.1 (0.05, 21.31)	0.015 (0.05, 0.14)	0.01 (0, 0.12)	0.02 (0.01, 0.15)	0.02 (0.01, 0.15)	0 (0, 0.005)	*P* < 0.001***
Number of comments	301 (2, 1082)	7 (3.5, 61)	0.5 (0, 2)	1 (0, 8)	2 (0, 67)	0 (0, 4)	*P* < 0.001***
Number of comments/day	1.73 (0.02, 6.09)	0.01 (0, 0.08)	0.01 (0, 0.1)	0.15 (0.01, 1.61)	0.03 (0.01, 0.22)	0.01 (0, 0.05)	*P* < 0.001***
Number of shares	262 (6930)	13 (2, 82)	1 (0, 6.5)	1 (0, 2)	10 (2, 126)	3 (1.5, 36.5)	*P* < 0.001***
Number of shares/day	1.32 (0.89, 4.61)	0.014 (0.02, 0.096)	0.002 (0, 0.003)	0.001 (0, 0.026)	0.024 (0.003, 0.277)	0.003 (0.001, 0.019)	*P* < 0.001***
**Quality Assessment**							
DISCERN reliability	17 (15, 19)	18 (14.5, 19)	20 (16.5, 25.5)^c^	19 (16, 20)	19 (18, 21)	18 (16.5， 19.5）	*P* = 0.006**
DISCERN treatment	11 (9, 14)	9 (7.5, 10.5)	9.5 (8, 16.5)	9 (8, 12)	11 (9, 22)	12 (10, 15.5)	*P* = 0.078
DISCERN quality	2 (2, 3)	2 (1, 2)	2.5 (2, 3.75)	2 (2, 3)	2 (2, 3)	2 (1.5, 2)	*P* = 0.061
DISCERN total	29 (27, 34)	29 (23, 31.5)	32 (29, 45.5)	30 (26, 34)	32 (29, 48)	32 (31, 34)	*P* = 0.083
JAMA score	2 (2, 3)	2 (2, 2)	3 (3, 3)^d^	2 (2, 3)	3 (2, 3)	2 (1.5, 2)	*P* = 0.001**

a: All results were given as frequency and percentages.

b: Kruskal-wallis H test was used.

c-d: Compared with independent medical professionals, patients or guardians and independent non-medica users, *P* < 0.001, respectively.

*: *P* < 0.05, **: *P* < 0.01, ***: *P* < 0.001.

**Fig. 3:**
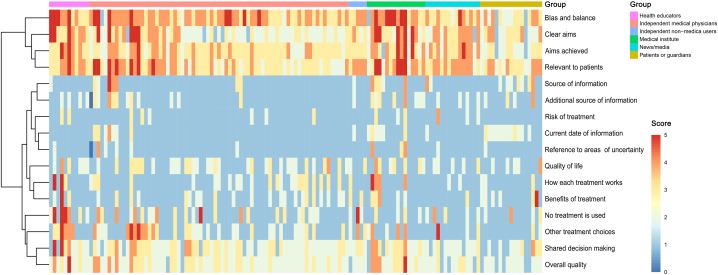
**DISCERN score of the videos.** Unsupervised hierarchical clustering was conducted based on DISCERN score items (rows) and individual videos (columns, n = 135). The categorical item scoring ranges from 1 (not addressed/fulfilled) to 5 (fully addressed/fulfilled), and the video group is indicated in the top row of the heatmap.

**Fig. 4:**
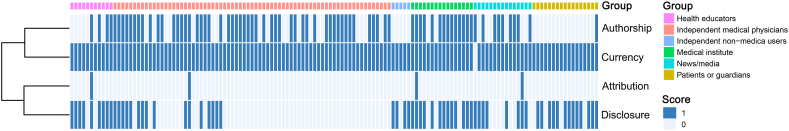
**JAMA score for videos.** Unsupervised hierarchical clustering was performed based on JAMA score items (rows) and single videos (columns, n = 135). The categorial item scoring is either 1 (addressed/fulfilled) or 0 (not addressed/fulfilled), and the video group is represented in the top row of the heatmap.

Further analysis showed that videos uploaded by independent medical professionals had significant correlations with the DISCERN reliability scores (*P* = 0.006) and JAMA scores. Data revealed that videos uploaded by medical institutes (median (IQR): 20 (16.5, 25.5)), health educators (median (IQR): 19 (18, 21)), and news/media (median (IQR): 19 (16, 20)) had higher DISCERN reliability scores than those by independent medical professionals (median (IQR): 19 (17, 21)). Similarly, the JAMA score for both medical institutes (median (IQR): 3 (3, 3)) and health educators (median (IQR): 3 (2, 3)) was higher than that for independent medical professionals (median (IQR): 2 (3, 2)). However, there were no significant differences in DISCERN treatment scores, quality scores, or total scores among the various video sources (Fig. 3). Unsupervised hierarchical clustering revealed that very few videos achieved high scores, especially in questions related to risk, benefits, treatment mechanisms, source, information date, and reference to areas of uncertainty. After unsupervised hierarchical clustering, detailed results of the JAMA score, as well as the divided groups by video source shown in Fig. 4. The specific criteria for currency were met in the vast majority of videos (98%). However, only a few videos satisfied the criteria of attribution (8%). It is noteworthy that videos uploaded by patients or guardians often lacked authorship, and over 70% of videos by independent medical professionals failed to disclose potential conflicts of interest.

### 3.2 Distribution of DISCERN classification

The data concerning the distribution of DISCERN classifications revealed that 19.3% of the videos fell into the “very poor" category, while 67.4% were categorized as “poor." Additionally, 8.9% were classified as “fair," 3.7% were rated as “good," and only 0.7% achieved the distinction of “excellent" (as indicated in Table 3). Furthermore, it's noteworthy that the duration of videos in the “good" and “excellent" categories was longer than those in the other classifications (*P* < 0.001). However, there were no significant differences between the groups in terms of any other video characteristics.

**Table 3:** Distribution of DISCERN classification according to video characteristics and source.

Variable	Very poor	Poor	Fair	Good	Excellent	*P* value^a^
Video characteristics (IQR)						
Duration (s)	64.5 (41.75,95.5)	77 (58,169)	184.5 (109.5, 366.5)	314 (220, 2293)	284	*P* < 0.001***
Number of days online	162 (43.25,525.25)	296 (132, 527)	115.5 (33.25,510.75)	238 (115, 823)	208	*P* = 0.389
Number of likes	27 (2.75, 1977.75)	44 (1, 2352)	9 (2.5, 20.5)	10 (4.5, 43.5)	26	*P* = 0.406
Number of likes/day	0.17 (0.01, 11.485)	0.13 (0.01, 6.66)	0.045 (0.013, 0.158)	0.02 (0.005, 0.195)	0.13	*P* = 0.474
Number of comments	65 (0, 436.75)	39 (0, 715)	1.5 (0, 2.75)	2 (0, 34.5)	7	*P* = 0.100
Number of comments/day	0.065 (0.001, 3.928)	0.06 (0.001, 2.18)	0 (0, 0.035)	0 (0, 0.145)	0.03	*P* = 0.198
Number of shares	23.5 (0.75, 384)	50 (1, 513)	2 (1, 9.5)	14 (3.5, 76.5)	2	*P* = 0.083
Number of shares/day	0.08 (0.001, 2.318)	0.117 (0.003, 1.88)	0.012 (0.002, 0.028)	0.026 (0.012, 0.282)	0.01	*P* = 0.116
**Video source (N, %)**						
Independent medical Professionals	14 (10.4%)	50 (37%)	6 (4.4%)	1 (0.7%)	0 (0%)	
Patients or guardians	5 (3.7%)	11 (8.1%)	1 (0.7%)	0 (0%)	0 (0%)	
Medical institute	3 (2.2%)	8 (5.9%)	2 (1.5%)	2 (1.5%)	1 (0.7%)	
News/media	4 (3%)	10 (7.4%)	1 (0.7%)	0 (0%)	0 (0%)	
Health educators	0 (0%)	7 (5.2%)	2 (1.5%)	2 (1.5%)	0 (0%)	
Independent non-medica users	0 (0%)	5 (3.7%)	0 (0%)	0 (0%)	0 (0%)	
Total	26 (19.3%)	91 (67.4%)	12 (8.9%)	5 (3.7%)	1 (0.7%)	

a: Kruskal-wallis H test was used.

*: *P* < 0.05, **: *P* < 0.01, ***: *P* < 0.001.

### 3.3 Video characteristics on different social media platforms

**Fig. 5:**
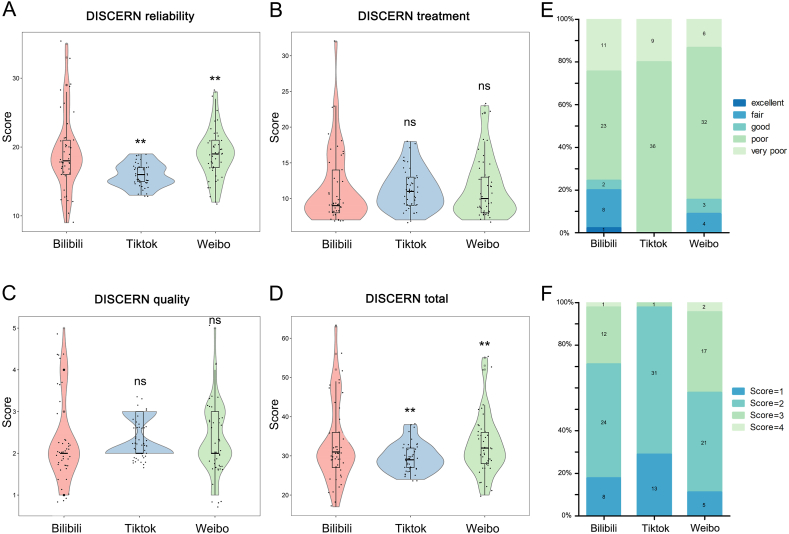
**Video characteristics on different social media platforms.** (A–D) DISCERN score comparison of videos on different social media platforms. (E) Distributions of DISCERN classification. (F) Distributions of JAMA scores.

Ultimately, this study included 45 videos each from TikTok, Bilibili, and Weibo, allowing us to conduct a more in-depth analysis. It was observed that videos uploaded on Bilibili had the longest duration (median (IQR): 199 (94, 407)) and were online for the greatest number of days (median (IQR): 525 (122.5, 934.5)). In contrast, TikTok videos had the shortest duration of being online (median (IQR): 65 (48.5, 91.5)). Interestingly, videos on Bilibili seemed to garner more “likes" (median (IQR): 1658 (1207, 6185)), “comments" (median (IQR): 823 (316.5, 1537.5)), and “shares" (median (IQR): 664 (277.5, 1405)) compared to TikTok and Weibo (*P* < 0.001; Table 4). Furthermore, Bilibili videos obtained a higher DISCERN reliability score compared to Weibo and TikTok (Fig. 5A), while there were no differences in DISCERN treatment and quality (Fig. 5B and C). The median DISCERN total score for videos on Bilibili was also higher than that of Weibo and TikTok (Fig. 5D). The median DISCERN treatment score and quality exhibited no significant differences between the three social media platforms. For a more detailed breakdown of DISCERN classification and JAMA scores, please refer to Fig. 5E and F.

**Table 4:** Video characteristics of included videos in different social media platforms.

Variables	TikTok	Bilibili	Weibo	*P* value^a^
Numbers of videos (N, %)	45 (33.3%)	45 (33.3%)	45 (33.3%)	–
Duration (s)	65 (48.5, 91.5)^b^	199 (94, 407)	72 (52.5, 164)	*P* < 0.001***
Number of days online	267 (91.5, 400) c	525 (122.5, 934.5)	199 (58.5, 444)	*P* < 0.001***
Number of likes	1658 (1207, 6185) d	12 (4, 39.5)	1 (0.5, 3)	*P* < 0.001***
Number of likes/day	10.95 (5.12, 58.235) e	0.03 (0.01, 0.14)	0.001 (0, 0.045)	*P* < 0.001***
Number of comments	823 (316.5, 1537.5) f	2 (0, 7)	0 (0, 4.5)	*P* < 0.001***
Number of comments/day	3.21 (1.765, 13.32) g	0.003 (0, 0.045)	0 (0, 0.01)	*P* < 0.001***
Number of shares	664 (277.5, 1405) h	7 (1.5, 26.5)	1 (0, 6.5)	*P* < 0.001***
Number of shares/day	2.66 (1.33, 12.315) i	0.019 (0.003, 0.054)	0.003 (0, 0.019)	*P* < 0.001***

a: Kruskal-wallis H test was used.

b, c: Compared with Bilibili, *P* < 0.001, respectively.

d-i: Compared with Bilibili and Weibo, *P* < 0.001, respectively.

*: *P* < 0.05, **: *P* < 0.01, ***: *P* < 0.001.

### 3.4 Correlation of factors influencing of DISCERN score and JAMA score

To minimize the variability in our assessment results, we employed both the DISCERN and JAMA instruments for evaluating video quality. Spearman's test uncovered correlations between factors influencing DISCERN scores and JAMA scores. The analysis revealed a significant positive correlation between the DISCERN total score and the video duration (r = 0.417, *P* < 0.001). Furthermore, the JAMA score also exhibited a significant positive correlation with the video duration (r = 0.223, *P* = 0.009). Notably, the DISCERN total score was significantly connected with the JAMA score (r = 0.558, *P* < 0.001; Table 5).

**Table 5:** The factors influencing of DISCERN score and JAMA score were tested for correlation.

Variables	DISCERN score		JAMA score	
	r	*P* value^a^	r	*P* value^a^
DISCERN score	**-**	**-**	0.558	*P* < 0.001***
JAMA score	0.558	*P* < 0.001***	**-**	**-**
Duration(s)	0.417	*P* < 0.001***	0.223	*P* = 0.009
Number of days online	0.050	*P* = 0.568	0.118	*P* = 0.174
Number of likes	0.001	*P* = 0.988	0.007	*P* = 0.931
Number of likes/day	0.015	*P* = 0.866	0.069	*P* = 0.428
Number of comments	0.019	*P* = 0.828	0.012	*P* = 0.886
Number of comments/day	0.009	*P* = 0.916	0.077	*P* = 0.373
Number of shares	0.027	*P* = 0.754	0.023	*P* = 0.790
Number of shares/day	0.032	*P* = 0.712	0.055	*P* = 0.528

a: Spearman's test was used.

*: *P* < 0.05, **: *P* < 0.01, ***: *P* < 0.001.

## 4 Discussion

### 4.1 Motivation and implications of this study

With the development of the internet and the surging popularity of social media, these platforms have become a major source of entertainment and information for people, garnering substantial attention. As vast repositories of information, various social media platforms have emerged as pivotal channels for disseminating health-related information and providing the general population with access to medical and health knowledge. Notably, platforms like TikTok, Bilibili, and Weibo offer a more direct and convenient means of access to such information for the people of China. However, prior research has highlighted a concerning trend: approximately two-thirds of medical videos are deemed unsatisfactory, with one-third containing inaccuracies [18,33]. This seriously affects the correctness and effectiveness of health information dissemination to the public. The motivation behind this study stems from personal experiences, as the author was confronted by questions from both relatives and patients who pointed out inconsistencies between information they found on platforms like Bilibili and TikTok and the information the author provided. These interactions sparked the author's contemplation and served as a source of inspiration. The information gleaned from videos on social media platforms can be likened to a double-edged sword, posing both opportunities and concerns.

Previous studies have shown that information targeted at pregnant women on social platforms is readily accessible and garners significant views, but it is often characterized by low quality and reliability [34]. GDM, as one of the most prevalent pregnancy complications, is associated with adverse pregnancy outcomes [35,36]. It is no wonder that high-quality care and management are crucial for women with GDM to benefit pregnancies [37]. Consequently, it becomes essential to conduct a quantitative assessment of internet videos in China specifically pertaining to GDM, a task that has not been previously undertaken.

### 4.2 Major findings

Upon conducting a thorough assessment using DISCERN and JAMA, it became evident that the quality of all types of videos was consistently low. The mean scores of DISCERN and JAMA were 31.84/75 and 2.33/4, respectively. In terms of DISCERN classification, a significant 86.7% of the videos fell into the “poor" or “very poor" quality categories, with merely 4.4% being categorized as “good" or “excellent." While most videos had clear objectives and successfully achieved their goals, over two-thirds of the videos lacked critical information such as video source attribution, current upload dates, and detailed treatment information. Furthermore, more than half of the videos failed to provide comprehensive treatment descriptions, including explanations of how treatments work and their associated benefits. These findings underscore the low quality and lack of reliability in these videos. JAMA scores showed that almost more than 90% of videos have currency, but only four videos contributed the attribution to the public. What worries people about the video quality on social platforms for the first time is that Keelan et al. found that 38% of analyzed YouTube videos were against vaccination, but the average star rating and views were higher than those supporting vaccination [38]. This observation has led to a growing body of research focused on the subpar quality of videos on social media platforms [19].

The majority of videos were uploaded by authors from Henan, Beijing, and Guangdong, located in the south, north, and middle of China, respectively. It remains a question whether the number of videos is associated with regional distribution or regional development, a topic that warrants further discussion. Information from video sources manifested that more than half videos were uploaded by independent medical professionals, but the total quality is poor. Although the videos uploaded by independent medical professionals had less duration, they were likely to receive more public attention, such as likes, comments, and shares, which was consistent with the results of previous similar studies [39,40]. Owing to most professionals were fond of dividing videos into multiple sections and uploading a series of videos or focusing on one of the related topics to share. Moreover, such results are worth our worrying and consideration: the quality of health information provided by experts does not meet the expectations of the public and needs to be improved.

Many viewers tend to initially select videos with higher popularity, anticipating reliable and pertinent information from specialists. However, Xue et al. revealed a disconcerting fact: among their sample of 61 analyzed videos, over 33% contained unequivocal misinformation [23]. To make matters worse, a positive correlation between the presence of misinformation and the number of views. Previous studies pointed out that popularity did not necessarily equate to higher quality, and higher-quality videos were not consistently among the most popular ones [24]. Consequently, the number of likes, comments, and shares a video received from the public did not consistently correlate with its quality or source. It was observed that videos with longer durations and more detailed information tended to receive a higher DISCERN classification. In contrast, TikTok had the shortest duration, but with the most popular and lower DISCERN classification, which suggested the number of likes, comments, and shares were not related to the DISCERN classification. It highlights the fact that the public possesses a certain degree of discernment and does not always unquestioningly embrace high-quality health information on social media platforms. These findings indicated the significance of the ability of public self-identification and the level of health self-education. Eventually, spearman's tests showed the DISCERN scores had a significant correlation with JAMA scores, emphasizing the pivotal role and scientific validity of the DISCERN and JAMA instruments in the evaluation of these videos. Furthermore, this showed the authors used the DISCERN and JAMA instruments correctly to evaluate the videos through their expertise.

### 4.3 Expectations

GDM is among the obstetrical complications that have the most adverse impact, particularly when coupled with other complications [41,42]. Social media platforms are increasingly playing a critical role in public health engagement. However, the quality of videos on these platforms often falls short [43]. Therefore, it is essential for video uploaders, especially medical professionals, to offer a more comprehensive and accurate portrayal of information while avoiding any misleading or incomplete content. Based on the findings of our study, we recommend that video uploaders proactively include vital details such as the video's source, the date of its upload, and comprehensive treatment information. Additionally, social media platforms themselves bear a regulatory responsibility for ensuring the quality of videos available to the public. It is incumbent upon us to provide constructive suggestions for the use of social media platforms and encourage government involvement in the regulation of health-related information on these platforms, to a reasonable extent.

### 4.4 Limitations

Although our study is the first to investigate the content and quality of GDM-related videos on social media platforms in China, it does come with a few limitations. Firstly, we only considered videos from China, potentially overlooking valuable content in other languages. Although our primary focus was on Chinese videos, it's worth acknowledging that videos in other languages may offer more high-quality information and a more comprehensive perspective. Secondly, despite using new accounts for our searches, it's important to note that search algorithms can yield different results based on factors like location, user behavior, and other unknown variables in a dynamic system. Finally, this study focused on the most frequently used and most popular social media platforms, but the contribution of other platforms to public health information remains to be investigated.

## 5 Conclusion

In conclusion, this study evaluated the information quality of 135 GDM-related videos on Chinese social media platforms. The findings revealed that the majority of these videos were uploaded by independent medical professionals, but regrettably, their overall quality was lacking. Of particular concern is the fact that these videos often offered incomplete and potentially misleading information, particularly in the context of treatment. It is imperative to enhance collaboration with professionals and social media platforms and enhance the quality of GDM-related videos. The public should exercise caution when seeking GDM-related information on these platforms and actively promote their self-education.

## Ethics approval and consent to participate

Not applicable.

## Funding

This work was funded by the Program for Youth Innovation in Future Medicine, 10.13039/501100004374Chongqing Medical University (grant numbers W0068).

## Data availability statement

The data underlying this article will be provided by the corresponding author upon reasonable request.

## Consent for publication

Not applicable.

## CRediT authorship contribution statement

**Qin-Yu Cai:** Writing – original draft, Visualization, Validation, Software, Methodology, Investigation, Formal analysis, Data curation, Conceptualization. **Jing Tang:** Validation, Software, Methodology, Investigation, Formal analysis, Data curation, Conceptualization. **Si-Zhe Meng:** Visualization, Software, Methodology, Formal analysis, Data curation, Conceptualization. **Yi Sun:** Software, Methodology, Investigation, Data curation, Conceptualization. **Xia Lan:** Writing – review & editing, Validation, Supervision, Resources, Project administration, Methodology, Conceptualization. **Tai-Hang Liu:** Writing – review & editing, Supervision, Project administration, Methodology, Funding acquisition, Data curation, Conceptualization.

## Declaration of competing interest

The authors declare that they have no known competing financial interests or personal relationships that could have appeared to influence the work reported in this paper.
