# Supplementary material for: Quality assessment of videos on social media platforms related to gestational diabetes mellitus in China: A cross-section study
Source: Heliyon. 2024 Mar 31;10(7):e29020. doi: 10.1016/j.heliyon.2024.e29020 (PMC11015130; doi:10.1016/j.heliyon.2024.e29020)
Supplement: Multimedia component 1 [file mmc1.docx]

| **Question** | | | **Score** |
| --- | --- | --- | --- |
| Section 1 | 1 | Are the aims clear? | 1-5 |
|  | 2 | Does it achieve its aims? | 1-5 |
|  | 3 | Is it relevant? | 1-5 |
|  | 4 | Is it clear what sources of information were used to compile the publication (other than the author or producer)? | 1-5 |
|  | 5 | Is it clear when the information used or reported in the publication was produced? | 1-5 |
|  | 6 | Is it balanced and unbiased? | 1-5 |
|  | 7 | Does it provide details of additional sources of support and information? | 1-5 |
|  | 8 | Does it refer to areas of uncertainty? | 1-5 |
| Section 2 | 9 | Does it describe how each treatment works? | 1-5 |
|  | 10 | Does it describe the benefits of each treatment? | 1-5 |
|  | 11 | Does it describe the risks of each treatment? | 1-5 |
|  | 12 | Does it describe what would happen if no treatment is used? | 1-5 |
|  | 13 | Does it describe how the treatment choices affect overall quality of life? | 1-5 |
|  | 14 | Is it clear that there may be more than 1 possible treatment choice? | 1-5 |
|  | 15 | Does it provide support for shared decision making? | 1-5 |
| Section 3 | 16 | Based on the answers to all of these questions, rate the overall quality of the publication as a source of information about treatment choices. | 1-5 |

**Supplementary Table 1**. **DISCERN Instrument Criteria.**

**Supplementary Table 2**. **JAMA** **benchmark criteria.**

| **JAMA Benchmarks** | **Explanation** | **Score** |
| --- | --- | --- |
| Authorship | Authors and contributors, their affiliations, and relevant credentials should be provided | 0 or 1 |
| Attribution | References and sources for all content should be listed clearly, and all relevant copyright information should be noted | 0 or 1 |
| Disclosure | Website “ownership” should be prominently and fully disclosed, as should any sponsorship, advertising, underwriting, commercial funding arrangements or support, or potential conflicts of interest | 0 or 1 |
| Currency | Dates when content was posted and updated should be indicated | 0 or 1 |

**Supplementary Table 3**. **Description of the Global Quality Score (GQS) 5-point scale used to evaluate videos.**

| **GQS** | **Description** |
| --- | --- |
| 1 | Poor quality; poor flow of the site; most information missing; not at all useful for patients |
| 2 | Generally poor quality and poor flow; some information listed but many important topics missing; of very limited use to patients |
| 3 | Moderate quality; suboptimal flow; some important information is adequately discussed but others poorly discussed; somewhat useful for patients |
| 4 | Good quality and generally good flow; most of the relevant information is listed, but some topics not covered; useful for patients |
| 5 | Excellent quality and excellent flow; very useful for patients |
